# Effect of streptozotocin-induced diabetes on left ventricular function in adult rats: an in vivo Pinhole Gated SPECT study

**DOI:** 10.1186/1475-2840-6-30

**Published:** 2007-10-15

**Authors:** Bernard Cosyns, Steven Droogmans, Caroline Weytjens, Tony Lahoutte, Guy Van Camp, Danny Schoors, Philippe R Franken

**Affiliations:** 1 Cardiology department, UZ Brussel,, Belgium; 2 Nuclear Medicine department, UZ Brussel, Belgium; 3 In Vivo Cellular and Molecular Imaging Center (ICMIC), VUB, Belgium

## Abstract

**Background:**

Recent studies have suggested that diabetes mellitus (DM) may cause left ventricular (LV) dysfunction directly resulting in increased susceptibility to heart failure. Using pinhole collimators and advances in data processing, gated SPECT was recently adapted to image the rat heart. The present study was aimed to assess this new imaging technique for quantifying LV function and remodeling from the Streptozotocin (STZ) rat model compared to controls.

**Methods:**

Twenty one rats were randomly assigned to control or diabetic group. Six months after the induction of diabetes by STZ, Pinhole 99 m Tc-sestamibi gated SPECT was performed for determining rat LV volumes and function. Post-mortem histopathologic analysis was performed to evaluate the determinant of LV remodeling in this model.

**Results:**

After six months, the normalized to body weight LV End-systolic volume was significantly different in diabetic rats compared to controls (0.46 ± 0.02 vs 0.33 ± 0.03 μL/g; p = 0.01). The normalized LV End-diastolic volume was also different in both groups (1.51 ± 0.03 vs 0.88 ± 0.05 μL/g; p = 0.001) and the normalized stroke volume was significantly higher in STZ-rats (1.05 ± 0.02 vs 0.54 ± 0.06 μL/g; p = 0.001). The muscular fibers were thinner at histology in the diabetic rats (0.44 ± 0.07 vs 0.32 ± 0.06 AU; p = 0.01).

**Conclusion:**

Pinhole 99 m Tc-sestamibi gated SPECT can successfully be applied for the evaluation of cardiac function and remodeling in STZ-induced diabetic rats. In this model, LV volumes were significantly changed compared to a control population, leading to a LV dysfunction. These findings were consistent with the histopathological abnormalities. Finally, these data further suggest the presence of diabetes cardiomyopathy.

## Background

Diabetic cardiomyopathy is known to develop in humans in the absence of coronary or hypertensive disease [1]. The mechanism by which diabetic cardiomyopathy develops has been studied in literature using in-vivo and ex-vivo experiments [2]. It has been postulated that endothelial dysfunction, endomyocardial fibrosis, direct toxic effect of hyperglycemia on cardiomyocytes and autonomic neuropathy play an important role. These cardiovascular complications compromise cardiac performance ultimately resulting in cardiac failure. A high prevalence of cardiac failure is seen in individuals with diabetic cardiovascular complications, with diabetic cardiomyopathy as one of the key determinants [3].

Because of its accurate reflection of human pathophysiology [4], the rat model has also been widely used to study LV remodeling. Recently, it has been reported that if the diabetes persists for a long enough time, it induces cardiomyopathy, manifesting decreased resting LV ejection fraction (LVEF) [5,6]. Different imaging modalities have been used to asses the onset and time course of LV function abnormalities in small animals with Streptozotocin (STZ) induced diabetes, especially in rats [7-13]. With the use of pinhole collimators and the advance in data processing, gated SPECT was recently adapted to image the rat heart [14]. The present study was aimed to assess this new imaging technique for quantifying LV function and remodeling from the STZ rat model compared to controls. In addition post-mortem histopathologic analysis was performed to evaluate the determinant of LV remodeling in this model.

## Methods

### Animals handling and study protocol

A total of 21 adult male Wistar Unilever rats (Harlan, The Netherlands) (11 weeks-old, 345 ± 62 g) were studied.

Diabetes mellitus was induced in 12 rats by a single intravenous injection of 45 mg/kg STZ in a 0.1 M citrate buffer solution. Three days after treatment with STZ, tail vein blood glucose samples were measured with Onetouch^R ^glucometer (Johnson & Johnson) to ensure induction of diabetes (glycemia > 200 mg/dL). In order to study the diabetic animals over a longer period, a half subcutaneous implant of insulin (2 U/24 h, Linplant^®^, Canada) was inserted at 2 and 4 months.

During all the study the animals were housed in stainless steel cages with sawdust bedding. They were kept at an average room temperature of 24°C, a relative humidity of 50% and a 12-hour day/night cycle. All rats had unlimited access to food and water during follow up.

This study was approved by the Animal Research Committee at the Vrije Universiteit Brussel and conformed to the American Heart Association Guidelines for use in animal research.

### Pinhole Gated SPECT Acquisitions

The animals were sedated by intraperitoneal injection of 60 mg/kg of sodium pentobarbital, and 99mTc-sestamibi (400–700 MBq in a 0.3- to 0.5-mL volume) was injected intravenously 40–60 min before starting gated SPECT acquisition. During the acquisition, the animals were in prone position and were connected to a standard electrocardiogram monitor by 3 electrodes placed on the inner surfaces of limb extremities.

As previously described [14], pinhole gated SPECT acquisitions were performed using a dual head γ-camera (e.cam™ Signature series Fixed 180; Siemens Medical Solutions inc.) equipped with a 3-mm pinhole collimator (195-mm focal length; 43-mm radius of rotation). A number of 128 projections of 30 seconds per step were acquired on a 360° rotation and with 16 frames per cardiac cycle. Additional acquisition parameters were as follows: 64 × 64 matrix, 2.0 zoom, 126- to 154-keV energy window, beat acceptance window set to ± 20% of averaged R-R interval. Total acquisition time was 21 min.

### Reconstruction and analysis of Pinhole Gated SPECT images

As already described [14-16], projection data were corrected for the pinhole geometry, the mechanical shift of the camera-head rotation and for the nonuniform sensitivity of pinhole detection. Images were thereafter reconstructed using an ordered-subset expectation maximization (OSEM) iterative process (2 iterations and 8 subsets), developed in our institution. In addition, a temporal Fourier filtering was incorporated after each iteration to reduce image noise [14-16]. Voxel size of the reconstructed images was 0.6 mm and the spatial resolution was 3 mm in the central slice when determined by the full width at half maximum on a point source of 99mTc.

**Figure 1:**
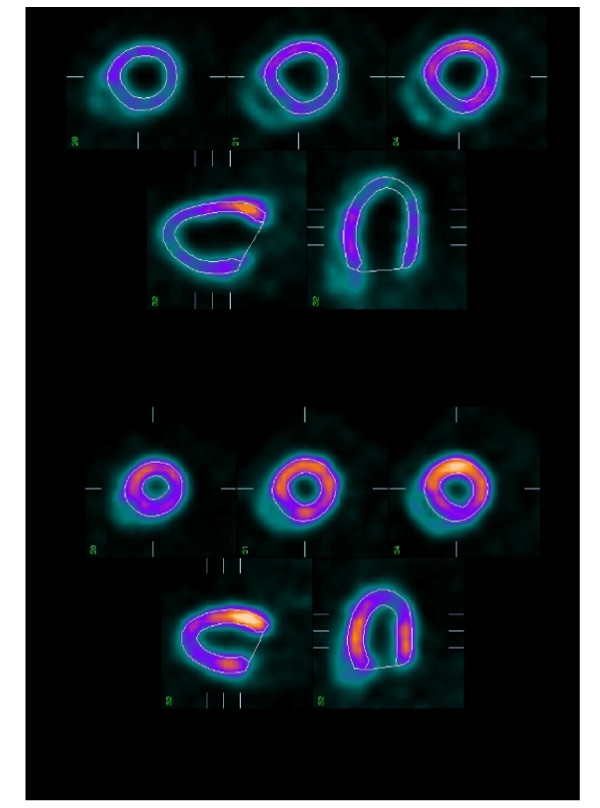
Example of pinhole SPECT images obtained in one diabetic rat from long-axis and short-axis slices in systole on the left and in diastole on the right.

Using the 4DMSPECT software (University of Michigan), myocardial perfusion polar maps were generated from the reconstructed short axis slices and used to quantify the extent of perfusion defects defined as the area below 2SD of a normal database obtained in 20 age matched normal rats. LVend-diastolic volume (EDV) and end-systolic volume (ESV), as well as LV ejection fraction (EF), were determined by QGS software (Cedars Sinai Medical Center, Los Angeles, USA) [17,18] on contiguous gated short-axis slices. Constraint limits of contour detection were applied only in case of evident errors with the fully automatic process. An example of Pinhole 99 m Tc-sesta mibi gated SPECT is presented in figure 1. The accuracy of LV volumes measured with Pinhole gated SPECT was previously validated with LV rat phantoms [19].

### Histology

At the end of follow-up, all rats were euthanized with high dose pentobarbital. The hearts were immediately removed and fixed in 4% neutral formalin for 2 hours. Three short-axis slices of myocardial tissue were obtained and embedded in paraffin. Heamatoxylin-eosin and Masson's trichrome staining were performed. Microscopy was done at 400× enlargement. Morphometry was performed by digital image analysis using a PC digital image camera (Digital Sight DS-5M, Nikon Corp, Japan) mounted on an Axiolab Zeiss light microscope (Carl Zeiss Corp, Germany) with a 10× objective (Acroplan, Zeiss). We used the NIH Image program (Image-J 1.35d, Nation Institutes of Health, Bethesda, USA). The program was calibrated with a graduated slide. Microscopic images were used to evaluate blindly the cardiomyocytes. The thickness of the muscular fibers was measured in every sample (10 measurements per sample). Color segmentation was applied to calculate the percentage of interstitial fibrosis.

### Statistical analysis

Data are expressed as mean ± standard error of the mean (SEM). Wilcoxon matched pairs test was used for paired series and Mann Whitney test was used for two-group unpaired comparisons. A value of p <0.05 was considered statistically significant.

## Results

### Clinical parameters

Glucometry and gravimetry parameters are summarized in Table 1. After six months, animals treated with STZ resulted in high glucose levels despite the administration of subcutaneous implant of insulin, when compared to controls animals (422 ± 52 vs 99 ± 12 mg/dl, p < 0.001). Other symptoms frequently associated with diabetic state such as lower body weights (399 ± 35 vs 658 ± 45 g; p < 0.0001), polyuria, polyphagia were observed in the diabetic rats. The heart mass decreased in the diabetic group but it was not significant compared to controls (1190 ± 280 vs 1370 ± 210 mg; p = 0.6). The heart/body weight ratio significantly increased in the diabetic group (2.9 ± 0.3 vs 2.2 ± 0.1 mg/g; p = 0.03). At the end of follow up there was a significant change in heart rate in the diabetic when compared to control rats (274 ± 9 versus 319 ± 11 bpm; p = 0.01).

**Table 1:** Glucometry and gravimetry data obtained at 20 weeks of diabetes

	Glycemia (mg/dL)	Body mass (g)	Heart mass (mg)	Heart to Body Mass ratio (mg/g)
Controls (n = 9)	99 ± 12	658 ± 45	1370 ± 210	2.2 ± 0.1
Diabetics (n = 12)	422 ± 52*	399 ± 35*	1190 ± 280	2.9 ± 0.3*

*p < 0.05

### Pinhole Gated SPECT measurements

After six months, derived from the pinhole Gated SPECT measurements, the normalized LVESV was significantly different in diabetic rats compared to controls (0.46 ± 0.02 vs 0.33 ± 0.03 μL/g; p = 0.01). The normalized LVEDV was also different in both groups (1.51 ± 0.03 vs 0.88 ± 0.05 μL/g; p = 0.001) and the normalized stroke volume was significantly higher in STZ-rats (1.05 ± 0.02 vs 0.54 ± 0.06 μL/g; p = 0.001). These results are summarized in Table 2.

**Table 2:** Left ventricular characteristics normalized to body mass

	Controls	Diabetics
End-Systolic Volume (μL/g)	0.33 ± 0.03	0.46 ± 0.02*
End-Diastolic Volume (μL/g)	0.88 ± 0.05	1.51 ± 0.03*
Stroke Volume (μL/g)	0.54 ± 0.06	1.05 ± 0.02*

* p < 0.05

The mean LVES (0.19 ± 0.01 vs 0.22 ± 0.02 ml; p = 0.117), LVED (0.59 ± 0.02 vs 0.58 ± 0.033 ml; p = 0.85) and SV (0.40 ± 0.01 vs 0.36 ± 0.02 ml; p = 0.2) were not significantly different between diabetic and control rats. The cardiac output (CO) was also not different in the diabetic and control groups (112.0 ± 5.62 vs 113.8 ± 5.49 ml/min; p = 1). Finally, there was no significant difference in perfusion scores between the two groups.

### Histology

**Figure 2:**
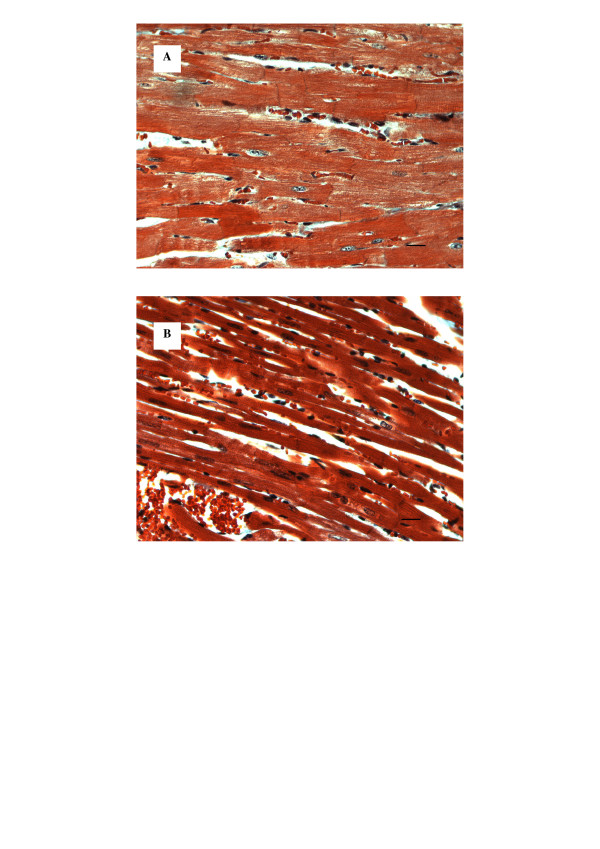
Histology of normal (a) and diabetic (b) myocardium (Heamatoxilin-eosin staining, 400×). The muscle fibers are thinner in the diabetic rats. Scale bar is 10 μm.

As shown in the figure 2, the muscular fibers were thinner in the diabetic rats (0.44 ± 0.07 vs 0.32 ± 0.06 AU; p = 0.01). However, there was no difference for interstitial fibrosis, first subjectively evaluated, in both groups. Therefore, color segmentation was applied to calculate the percentage of interstitial fibrosis. Percentage of fibrosis was less than 1% in both groups, with no difference between the two groups at the end of the follow up.

## Discussion

The study of cardiomyopathies in small animals may contribute to our understanding of the cardiac pathophysiology and to evaluate experimental treatment strategies. Therefore, this study was aimed to assess the role of Pinhole Gated SPECT for quantifying LV function and remodeling from the STZ rat model compared to controls. To the best of our knowledge, the present study is the first to show that this technique can successfully be applied for the non-invasive evaluation of cardiac function and remodeling in STZ-induced diabetic rats. Our results indicate that a significant increase in normalized EDV, ESV and SV had occurred after 6 months follow-up in STZ-induced diabetic group compared to normal group. Our data further indicate that these findings were consistent with the histological changes.

The STZ induced diabetic rats used in our experiments are reminiscent of a model of uncontrolled hyperglycemia due to the direct pancreatic beta cell destruction, and resulting insulin deficiency [20]. Our glucometry and gravimetry values (Table 1) are in accord with other published values at similar time points [21-23]. The significant differences in LV mass to body weight ratio between control and diabetic rats can be explained partly by the weight loss in the diabetic animals but we cannot exclude the presence of left ventricular hypertrophy. These rats with high dose of STZ resembled more like type 1 diabetes. Most of the previous small animals studies have used M-Mode echocardiography to document LV dysfunction and remodeling [5,6]. Despite its high temporal resolution, M-Mode analysis has many limitations and depends on a lot of geometrical assumptions. Probe alignment is a particularly important consideration for M-mode echocardiography, as the perpendicularity of the short axis plane with the ventricular walls is essential to validate the mathematical assumptions and equations used to derive fractional shortening, cardiac dimensions and LV mass parameters. Therefore, more reliable techniques are required. With the use of pinhole collimators and the advance in data processing, gated SPECT was recently adapted to image the rat heart [14]. This new technique allows the three-dimensional assessment of the full volumes of the heart, even in rodents. Pinhole 99 m Tc-sesta mibi gated SPECT has already been applied in the rat infarct model [24,25] and our pinhole gated SPECT technique was previously shown to provide precise and reproducible determinations of LV volume and LV function in normal adult rats[14]. The accuracy of LV volume measurements using cardiac phantoms has also been documented previously[19]. More recently, magnetic resonance imaging has been applied successfully in diabetic rats to evaluate the cardiac structures and function[12,13]. Although these studies have shown similar findings, the authors acknowledged that the LV planimetry was accomplished manually, limiting quantitative accuracy of this technique. Moreover, the use of 99 m Tc-sesta mibi gated SPECT reflects more the daily practice because of its broad availability.

There remains controversy regarding diabetes-induced LV dysfunction, especially in type 1 diabetes, in the absence of documented coronary artery disease. Some authors have been able to detect early systolic LV dysfunction en dilatation of the left ventricle in STZ induced diabetic cardiomyopathy [7-11]. On the contrary, others were not able to demonstrate a remodeling and a significant alteration of the systolic function in a similar rat population [26].

We observed an increase in mean normalized EDV of the diabetic group compared to controls. Meanwhile the normalized ESV of the diabetic rats also increased compared to controls but in lower proportion. As a consequence, the stroke volume increased in the diabetic group compared to controls. The significant increase of normalized EDV with diabetes in this study is in accordance with previous reports [5,23,27,28]. However, in contrast to our finding, other authors have shown no significant change or even an decrease in this parameter[12,29,30]. We also observed an increase in normalized ESV, suggesting a decrease in contractility, in accord with all previous studies [5,23,27,29,30]. The increase of the SV in diabetic rats was also shown by Bollano [27] when other authors suggested a decrease in SV[5,29,30]. This difference in SV could be explained by the significant bradycardia, that we and Bollano observed in the diabetic group. This was probably a compensatory mechanism to maintain cardiac output. The lower heart rate could be related to a longer exposure to hyperglycaemia. The diabetes duration, that may modify the time course of LV dysfunction in diabetic rats [5,23,31], was longer in our study (>20 weeks compared to 8–12 weeks in previous studies). The differences in LV volumes may be due to the method used to normalize the data or to the absence of normalization to body weight in other studies. Allometric relations exist between cardiac and body size measurements. However, the correct method to use in rat is unknown. We normalized LV mass to bodyweight as applied in the previous studies using the same animal model [23,28]. In addition, these differences may reflect the difference in the strain of rats (Wistar Unilever, Wistar Kyoto, Sprague-Dawley), since this factor has been shown to clearly influence cardiomyopathy in the STZ model of diabetes [32].

The presence of insulin implants (to permit a longer survival) had no impact on glucometry, gravimetric values and cardiac function. As shown by Litwin and colleagues, an insulin-therapy that aim to normalize the glycemia, also correct the cardiac abnormalities [31]. Finally, the dose of STZ injected (45 mg/kg) was low in this study. However, the impact on glucometry and gravimetric values was similar compared to the administration of higher dose of STZ, leading to equivalent absolute insulin deficiency.

In the present study, the muscular fibers were significantly thinner in the diabetic rats. This last finding was also consistent with previously published work [30]. These morphological changes may partially be responsible for the changes observed in LV remodeling observed in our study and the decrease in LV contractility. Nemoto et al have shown that a long-lasting diabetic state itself, but not the reduction of heart or body size, induces cardiomyocyte diameter reduction. The mechanism of the cellular remodeling of diabetic rats, that is, reduction of transverse diameter in cardiomyocytes remains unknown. These authors have suggested that insulin deficiency, which decreases protein synthesis and promotes protein degradation defective mitochondrial function or altered lysosomal enzyme activity might reduce the quantity of actin, resulting in cellular remodeling [30]. However, no other significant pathological changes were observed in our diabetic rats at the end of the follow up, especially the presence of fibrosis.

## Limitations

Our high dose STZ model resembled more like type 1 diabetes and therefore, our results may not be applied to other forms of diabetes. We did not study the right ventricle or the diastolic function. Finally, we did not use inotropic stimulation in order to unmask a decrease in the contractile reserve of the left ventricle.

## Conclusion

Pinhole 99 m Tc-sestamibi gated SPECT can successfully be applied for the non-invasive evaluation of cardiac function and remodeling in STZ-induced diabetic rats. In this model of diabetic rats, normalized LV volumes were significantly changed compared to a control population, leading to a LV dysfunction. This finding was consistent with the histopathological abnormalities. Finally, these data further suggest the presence of diabetes cardiomyopathy.

## Competing interests

The author(s) declare that they have no competing interests.
